# Perceived stress and monetary burden among thalassemia patients and their caregivers

**DOI:** 10.12669/pjms.344.15420

**Published:** 2018

**Authors:** Aliya Hisam, Najm us Sadiq Khan, Naseer Alam Tariq, Hira Irfan, Bushra Arif, Mawra Noor

**Affiliations:** 1 Dr. Aliya Hisam, MBBS, MPH, FCPS (Community Medicine). Associate Professor, Department of Community Medicine, Army Medical College, National University of Medical Sciences, Rawalpindi, Pakistan; 2 Dr. Najm us Saqib Khan, MBBS, MCPS, M.Sc., MBA, MBA, M.S, MPH, Assistant Professor, Community Health, Bahria Medical and Dental College, Islamabad, Pakistan. Department of Community Medicine, Army Medical College, National University of Medical Sciences, Rawalpindi, Pakistan; 3 Dr. Naseer Alam Tariq, MBBS, MSc (Med. Adm), MPH. Assistant Professor, Department of Community Medicine, Army Medical College, National University of Medical Sciences, Rawalpindi, Pakistan; 4 Dr. Hira Irfan, MBBS. House Officers Combined Military Hospital, Rawalpindi, Pakistan; 5 Dr. Bushra Arif, MBBS. House Officers Combined Military Hospital, Rawalpindi, Pakistan; 6 Dr. Mawra Noor, MBBS. House Officers Combined Military Hospital, Rawalpindi, Pakistan

**Keywords:** Caregivers, Cost, Monetary burden, Patients, Psychological burden, Stress, Thalassemia

## Abstract

**Objective:**

To find the perceived stress level and monetary burden in the thalassemia patients and their caregivers related to thalassemia treatment sessions.

**Methods:**

A descriptive cross sectional study was conducted at Rawalpindi Thalassemia Centre and Military Hospital Rawalpindi of six months’ duration from November 2016 to April 2017. A total of 87 sample size was calculated by using WHO sample size calculator. Participants were inducted through purposive sampling technique from thalassemia centers. A validated standardized Cohen’s Perceived Stress score was used. Data were entered and analyzed in SPSS 22.

**Results:**

Mean age of the participants was 30.42 ± 14.53 years. There were 30 (34.2%) males and 57 (65.8%) females. There were 39 (51.3%) patients and 48 (48.7%) caregivers. The mean income per month of the participants was 48706.9 ± 39492.68 PKR. The mean total expenditure per treatment session was 48706 ± 4037.12 PKR. Among the patients, there were 15 (38.5%) who were having average stress, while 4 (10.3%) were having moderate stress while 45 (51.7%0 were having severe stress. Among the caregivers, 10 (20.8%) were having mild stress, 13 (27.1%) were having moderate stress while 25 (52.1%) were having severe stress. The stress among the two groups was not statistically significant (p=0.066).

**Conclusion:**

More than half of the patients and caregivers were having a high perceived stress levels and there was a significant association between the two groups. The monetary burden was a lot to bear by the patients and the cost of treatment session most expensive.

## INTRODUCTION

Thalassemia is a haemoglobinopathy and specifically an autosomal recessive disorder. The word thalassemia is derived from the Greek word thalassa which means “sea”, and Latin word - emia (from Greek haema) meaning “blood”.^1^ Thalassemia syndromes are a group of disorders resulting from inherited mutations which lead to decrease synthesis of either α or β globin chains. These α and β globin chains along with iron containing heme group form the structure of adult hemoglobin HbA (α2β2). Because of the imbalance in synthesis of globin chain effects like anemia, hemolysis of red blood cells and tissue hypoxia are observed.^2^

In a normal person there is coordinated synthesis of the α- and β-globin chains, that is for each α-globin chain, a β-globin chain will be present consequently normal hemoglobin α2β2 (HbA) will be formed. However in thalassemia there is defective synthesis of either the α- or the β-globin chain.^3^ Considering the importance of prevention of haemoglobinopathies WHO has declared their control especially that of β-thalassemia its priority in developing world. The α and β- thalassemia are yet the most common single gene inherited disorders with soaring frequency in malarial endemic areas.^4^

Thalassemia though a preventable disease has an alarmingly high global prevalence. Prevalence of α-thalassemia in various region is: America 0-5%, Europe 1-2%, Eastern Mediterranean 0-2% and South East Asia 1-30% while prevalence of β-thalassemia is: America 0-3%, Eastern Mediterranean 2-18% and South East Asia 0-11%.^5^ Prevalence specifically of Pakistan though not properly documented is about 6%.^6^ It is estimated that 5000-9000 children with β-thalassemia are born per year and estimated carrier rate is 5-7% with 9.8 million carriers in total population.^7^

The disease itself has high morbidity and mortality which is augmented by the high frequency of treatment. Prevalence of complications is: Heart Failure 6-8%, Arrhythmias 5-7%, Hypogonadism 54.7%, Hypothyroidism 10.8%, Diabetes 6.4%, HIV infection 1.7% and thrombosis 1.1%.^8^ Another study in Northern Taiwan showed that among living patients over 15 years of age Hypogonadotropic hypogonadism, HCV infection, diabetes, heart failure and arrhythmias are common complications whereas the main causes of death are Heart disease, Bone Marrow Transplant related death and infections.^9^

**Fig.1:**
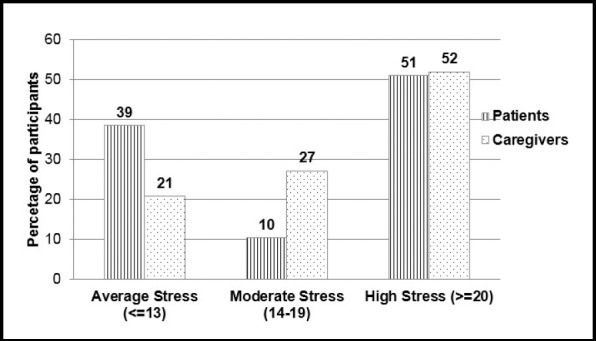
Stress among thalassemia patients and their caregivers (n=87) (p=0.066)**

**Fig.2:**
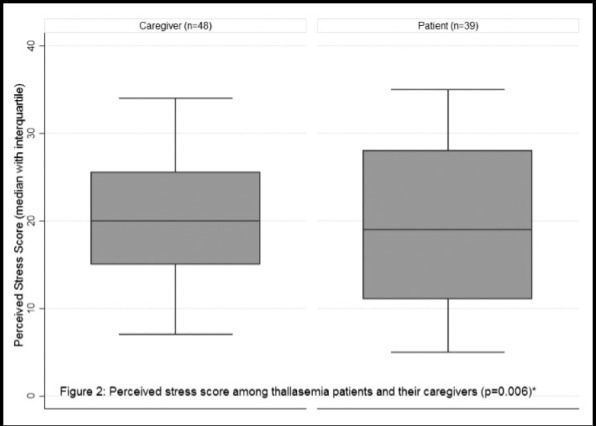
Perceived stress score among thalassemia patients and their caregivers (p=0.006)*

Stress can be defined as any uncomfortable or agonizing emotional experience associated with changes in behavior as well as biochemical and physiological changes or in simple words we can say it is a feeling of being worried or overwhelmed. It can affect anyone regardless of age or gender of person. Stress can result in both physical as well as psychological health issues.^10^

Stress in thalassemia patients and their parents can be attributed to a number of reasons like frequent treatment procedures and hospital visits, decreased life expectancy, expected complications from disease or treatment procedures and the monetary burden on parents/guardian.^11^ Also the long painful treatment sessions, such as to remove iron (iron overload being a complication of frequent blood transfusions) an eight hour long painful procedure (injecting chelators by chelation pump) is carried out, add to the stress factor.

Considering the fact that thalassemia is not a curable disease thus all that can be done is to improve the quality of life of the patient and decrease the morbidity (prevention being the vital control factor). Stress both in patients and their caregivers, which in our setup are mainly parents, is the biggest hindrance in improving the quality of life along with monetary issues. The aim of this study was to explore in which category of stress the thalassemia patients and their caregivers are? This will in the long run help policy makers, clinician and community to focus on stress and the monetary burden among thalassemia affected families and thus appropriate interventions can be planned and executed in this neglected but grave aspect of disease.

## METHODS

This was a descriptive cross sectional study conducted in Rawalpindi Thalassemia Centre and Military Hospital Rawalpindi. The duration of study was six months i.e. November 2016 till April 2017. A sample size of 87 was calculated by using Openepi info with hypothesized % frequency of thalassemia in population (p=1000) as 6% and with confidence limits as 5%. Non-probability purposive sampling was employed for the study. Thalassemia patients and their caretakers who usually assisted them for their treatment sessions and were responsible for their management and daily activities were included while those patients and caregivers who didn’t give consent, patients having any co-morbid conditions were excluded. This research was approved by ethical review committee of Army Medical College and informed voluntary consent was taken before starting interview and no risk or harm was anticipated to the best of our knowledge. Trained 4^th^ Year MBBS students after taking informed consent collected the data from the two centres. Data were collected from patients during the blood transfusion period. The data collection tool was a closed ended questionnaire. The questionnaire was translated into Urdu and verified by two subject specialists. It had two parts, first part was related to participant’s demographic details and monetory burden in terms of Pakistani rupees.

The participant were divided into two group i.e patients and their caregivers. The second part was related to stress evaluation by validated standardized Cohen Perceived Stress question aire.^12^ Cohen perceived stress questionnaire has 10 questions. Each question was rated on a 5-point scale ranging from never (0) to almost always (4). Positively worded questions were reverse scored, and the ratings were summed, with higher scores indicating more perceived stress. PSS-10 scores were obtained by reversing the scores on the four positive questions: For example, 0=4, 1=3, 2=2, etc. and then summing across all 10 items. Items 4, 5, 7, and 8 were taken as positively stated items. A score of 13 or less showed mild stress, 14-19 moderate stress and a score of 20 or more was considered high stress. Data were entered and analyzed in SPSS 22 and STATA. Qualitative data was presented as frequencies and percentages. Quantitative data was presented as mean and standard deviation. Chi square test of significance was applied to find association between the groups and qualitative variables such as gender, educational level and stress category. Independent sample t-test was applied to see mean difference among the groups and the quantitative variables such as age, income and cost. A p-value of < 0.05 was considered statistically significant.

## RESULTS

The total number of participants were 87 people, out of which 39 were patients and 48 were caregivers. The mean age of the patient was 17.09 ± 4.4 years while of the caregivers was 41.25 ± 10.17 years with statistically significant difference (p<0.001). Out of 39 patients, 16 (41%) were males and 23 (59%) were females whereas out of 48 caregivers, 14 (29.2%) were males and 34 (70.8%) were females.

Regarding education status, among patients, there were 10 (11.5%) illiterate, 24 (27.6%) having primary education, 37 (42.5%) having secondary education and 16 (18.4%) having higher level education. Among the caregivers, 4 (10.3%) were illiterate, 14 (35.9%) having primary education, 19 (48.7%) having secondary education and 2 (5.1%) having higher level of education. There was a statistically significant association among the two group’s educational status (p=0.026).

The mean income per month of the participants was 48706.9 ± 39492.68 PKR. The mean total expenditure per treatment session was 48706 ± 4037.12 PKR. Out of the total expenditure, the treatment, transport, food, accommodation and others miscellaneous cost were 2257.99 ± 3905.2 PKR, 764.94 ± 818.68 PKR, 325.29 ± 3905.2 PKR, 28.74 ± 139.69 PKR and 256.32 ± 1012.56 PKR respectively. Details are shown in Table-I.

**Table-I:** Demographic variables of the participants.

Demographic Variables	Combined (n=87)	Patient (n=39)	Caregivers (n=48)	p-value
Age mean ± SD	30.42 ±14.53	17.09 ± 4.4	41.25 ± 10.17	<0.001*
Gender n (%)				0.247**
Male	•30 (34.5)	•16 (41)	•14 (29.2)	
•Female	•57 (65.5)	•23 (59)	•34 (70.8)	
Educational Status n (%)				0.026**
•Illiterate	•10 (11.5)	•4 (10.3)	•6 (12.5)	
•Primary	•24 (27.6)	•14 (35.9)	•10 (20.8)	
•Secondary	•37 (42.5)	•19 (48.7)	•18 (37.5)	
•Higher level	•16 (18.4)	•(5.1)	•14 (29.2)
Income per month in PKR Mean ± SD	48706.9 ± 39492.68			-
Total Expenditure per treatment session in PKR mean ± SD	4733.27 ± 4037.12			-
•Treatment	2257.99 ± 3905.209			-
•Transport	764.94 ± 818.68		
•Food	325.29 ± 3905.2		
•Accommodation	28.74 ±139.691		
•Others	256.32 ±1012.56		

* Independent sample t-test,

** chi square test of significance, Mean + SD: mean + standard deviation, PKR: Pakistani Rupees

Among the patients, there were 15 (38.5%) who were having mild stress, while four (10.3%) were having moderate stress 45 (51.7%) were having severe stress. Among the caregivers, 10 (20.8%) were having mild stress, 13 (27.1%) were having moderate stress while 25 (52.1%) were having severe stress. The stress among the two groups was not statistically significant (p=0.066). As shown in Fig.1. The perceived stress score among the patients was 18.95 ± 8.87 while among the caregivers was higher that is 20.17 ± 6.6 which was also statistically significant at p = 0.006, as shown in Fig.2.

## DISCUSSION

Thalassemia is a chronic disorder that causes illness in children and impacts their families, complications of invasive treatment procedures and monetary burden causes physical, mental, emotional and psychological suffering.^13^ Mean age of our patients was 17 years, it is comparable to studies conducted in Taiwan^14^ and Egypt.^15^ Mean age of our caregivers was 41.25 years, it is similar to researches conducted in Rawalpindi^16^, Bahawalpur^17^ and Greece.^18^ No significant association was seen between stress in relation gender of patients and caregivers in the study conducted in India^19^ and Greek transfusion center^6^, which is also similar to our results.

Monetary burden also adds to the stress experienced by caregivers and this monetary burden can be attributed to treatment, transport, stay, medicines and food expenditures.^20^ In developing countries the cost of treatment is either too expensive or not available and iron overload in pediatric thalassemia patients is a common finding.^21^ In our study, mean total expenditure per treatment session was PKR 4753.9 which is equivalent to PKR 5000 of research conducted in Multan^22^. However, international studies conducted in ITHACA, Italy cost per treatment session was PKR 10000^23^ which is double the cost as compared to our study. Higher income was a significant determinant of higher quality of life for the physical and mental health dimensions and is inversely proportional to the levels of stress among the participants.^24^

In our study perceived stress score of patients came out to be severe in 51.28%, moderate level of stress among 10.25% and mild stress in 38.46%.According to Greek Transfusion center, severe depressive symptoms were found in 17 patients (15%), anxiety symptoms in 11(9.6%) and stress symptoms in 35(30.2%).^6^

Parents of thalassemia patients have to face higher levels of distress which affects the lifestyle and coping style of caregivers. Psychiatric morbidities aren’t limited to the patients, in fact they extend to the caregivers as well.^12^ In our study among participants, severe perceived stress score was seen in 52.08% of caregivers however a study conducted in Hyderabad Pakistan, showed 29% caregivers had severe stress.^25^ The parents (caregivers) of Thalassemia children were found to have severe parental stress assessed by Parental Stress Scale (PSS) in study conducted in Rawalpindi.^26^ In our study 54% of patients had psychological distress which is comparable to study of Department of Physiology Islamic International Medical College Rawalpindi, Pakistan in which 67.5% of parents were psychologically burdened.^27^ Another study conducted in Choithram Hospital Indore, India showed that 60.6% of parents were moderate to severe stress^16^ while in our study 79.16% caregivers were suffering from moderate to severely stress. In a study conducted in Bahawalpur, Pakistan, 45% of caregivers were psychologically distressed.^17^ The status of education among caregivers had no influence on the levels of stress and our study also showed no such difference with level of education statistically.^15^

### Limitations of the study

This study was conducted only in two centers, Thalassemia Center Rawalpindi and Military Hospital RWP which limits its generalization. Purposive sampling technique was used for this study and so the study results does not depict a true representation of the whole population.

## CONCLUSION

About half of the thalassemia patients and their caregivers who participated in the study had severe perceived stress and stress was more marked in caregivers as compared to patients. The monetary burden related to thalassemia treatment session was too much to bear by the patients and their caregivers, treatment cost being most expensive.

## RECOMMENDATIONS

On the basis of results of this study, we recommend that a holistic approach should be taken in treatment of Thalassemia with special attention to the caregivers including their counselling. The health care practitioners and physicians should be mindful of the psychological and emotional implications of this disease on the parents and their caregivers. Parents and caregivers should be properly counseled and educated about the disease and their apprehensions should be addressed. If the need is felt, psychiatric referral should be considered for severely stressed patients and their caregivers.

As far as the financial aspect of the disease is concerned it is also a contributory factor in the stress experienced by the caregivers so maximum relief should be provided in this regard, in form of government subsidies and financial assistance by NGOs. Hospitals can play their part by offering subsidies on blood bags, medications and other possible compensation packages.

In addition to all this, efforts should also be directed towards prevention of thalassemia by measures like premarital screening of thalassemia minor trait and appropriate counseling in case of detection to decrease the incidence of the disease and consequential psychological and economic burden.

### Authors’ Contribution

**HA:** Designed and did statistical analysis & final editing of manuscript.

**NSK & TNA:** Conceived the study, designed methodology and manuscript writing.

**IH, AB & NM:** data collection, discussion writing and review of manuscript.
